# Warburg revisited: lessons for innate immunity and sepsis

**DOI:** 10.3389/fphys.2015.00070

**Published:** 2015-03-09

**Authors:** Anup Srivastava, Praveen Mannam

**Affiliations:** Pulmonary, Critical Care and Sleep Medicine, Yale University School of MedicineNew Haven, CT, USA

**Keywords:** sepsis, metabolism, mTOR, HIF1a, Warburg effect, oxidative phosphorylation, glycolysis, inflammation

Warburg effect was first described as a mechanism by which the tumor cells rely on glycolysis to power proliferation and biosynthesis (Hsu and Sabatini, 2008). The transcription factor, hypoxia-inducible factor–1α (HIF-1α), and the serine/threonine protein kinase, mammalian target of rapamycin (mTOR), are major drivers of this metabolic switch (Lu et al., 2002; Sun et al., 2011). In recent years a number of reports have shown that immune cells such as monocytes, macrophages, dendritic cell, and T cells utilize a similar mechanism during activation (Palsson-McDermott and O'Neill, 2013). It is known that activated monocytes/macrophages switch their core metabolism from oxidative phosphorylation to glycolysis (Rodríguez-Prados et al., 2010; Liu et al., 2012). This process is similar to the Warburg effect in tumor cells because the activated immune cells need a readily available source of energy for phagocytosis, oxidative burst, and biosynthetic precursors to divide and produce cytokines.

The mechanism of such metabolic switching remains unclear but a recent article by Cheng et al. sheds light on this crucial process (Cheng et al., 2014). They studied the mechanism by which monocytes acquire trained immunity (Figure 1). This is a process by which monocytes on initial stimulation undergo epigenetic programing by histone modification, leading to stronger gene transcription upon restimulation (Netea et al., 2011). Using a model of β-glucan exposure of macrophages and subsequent LPS stimulation they show that the macrophages undergo epigenetic upregulation of genes expressing mTOR and glycolysis, that are targets of the transcription factor HIF-1α. The β-glucan trained monocytes showed elevated aerobic glycolysis, reduced basal respiration rate and increased glucose consumption and lactate production. This was associated with a decrease in oxygen consumption and reduced mitochondrial capacity, consistent with a metabolic shift from oxidative phosphorylation to glycolysis. This process occurs through the activation of Akt-mTOR-HIF-1α pathway. Blocking of the mTOR-HIF-1α pathway by chemical inhibitors abrogated the trained immunity and indirect inhibition of mTOR by AICAR (an activator of adenosine monophosphate-activated protein kinase, AMPK) had similar effects. Significantly, myeloid specific HIF1α deficient mice were not protected in a model of β-glucan induced tolerance against *S. aureus* sepsis, indicating that this pathway is important for septic responses.

**Figure 1:**
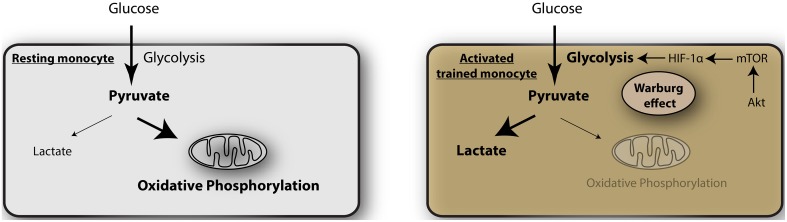
**Glycolysis as metabolic basis for trained immunity**. In resting monocytes the metabolism is predominated by oxidative phosphorylation, providing a majority of adenosine triphosphate (ATP) as the energy source. Activated monocytes during induction of trained immunity undergo a metabolic shift toward glycolysis, the Warburg effect, mediated by Akt-mTOR- HIF-1α.

This important study by Cheng et al. adds to a growing number of recent studies that have demonstrated that glycolytic flux and metabolic intermediates are drivers of immune responses. For example, macrophages exposed to LPS show a shift from oxidative phosphorylation to glycolysis (Rodríguez-Prados et al., 2010); and succinate, an intermediate in the TCA cycle, induced Interleukin 1beta (IL-1β) production (Tannahill et al., 2013). In addition, Glucose transporter 1 (Glut1) mediated increase in glycolysis drives a proinflammatory phenotype in macrophages (Freemerman et al., 2014). The realization that oncogenesis and immune responses have a common underlying mechanism of metabolic switching indicates that this fundamental biological process can be a potential drug target for many diseases. An important clinical application of the concept of metabolic switching is in the identification of therapeutic targets in sepsis.

Sepsis, a systemic inflammatory reaction to infection, is the leading cause of death in the world. The incidence of sepsis worldwide is 18 million every year with 30% mortality. The economic impact of sepsis is substantial with costs of up to $50,000/patient and $17 billion annually in United States alone (Slade et al., 2003; Moss, 2005). While in most cases we can control the infection with antibiotics, we lack therapies to minimize the hyperactive inflammatory response and downstream target injury (Lyle et al., 2014). Higher levels of lactate (product of glycolysis) and its slower rate of clearance during resuscitation predicts worse outcome in sepsis (Garcia-Alvarez et al., 2014). It is commonly thought that the lactate elevation is secondary to poor perfusion or microcirculatory disturbances, but it appears from recent studies that the lactate is more than just a marker of circulatory abnormalities and likely indicates a fundamental shift in metabolism to a more proinflammatory glycolysis. In line with this concept, a recent study has shown that pyruvate kinase M2- HIF-1α mediated Warburg effect drives mortality in sepsis (Yang et al., 2014). Our data on macrophages indicates that preservation of mitochondrial function under inflammatory conditions leads to attenuated cytokine responses (Srivastava et al., 2015). The mechanism by which metabolic switching regulates inflammatory processes is a novel and promising area of research. By targeting the upstream events in the inflammatory cascade we may get effective therapies for sepsis as well as for other inflammatory disorders.

## Conflict of interest statement

The authors declare that the research was conducted in the absence of any commercial or financial relationships that could be construed as a potential conflict of interest.
